# A functional role of the SNP -592 of human *IL10* gene regulatory region is associated with an increased IL-10 expression and risk for human papillomavirus cervical lesion and cervical cancer development

**DOI:** 10.1186/1753-6561-6-S6-P27

**Published:** 2012-10-01

**Authors:** K Torres-Poveda, A Burguete-García, M Bahena-Román, E Ortíz-Flores, K Delgado-Romero, D Cantú, A García-Carrancá, V Madrid-Marina

**Affiliations:** 1 Chronic Infection Diseases and Cancer Division, Centro de Investigación sobre Enfermedades Infecciosas, Instituto Nacional de Salud Pública, Cuernavaca, Morelos, Mexico; 2 Center for health of woman (CAPASAM), Health Services of State of Morelos, Cuernavaca, Mexico; 3 Unit of Biomedical Research in Cancer, Instituto Nacional de Cancerología/Biomedical Research Institute, National Autonomous University of Mexico, Mexico City, Mexico

## Background

An immunosuppressive state had been identified in women with persistent infection by the human papillomavirus (HPV), characterized by high levels of interleukin (IL)10 at cervix level [1]. The present study analyzed the association of SNPs in the regulatory region of the *IL10* gene with the risk of developing squamous intraepithelial lesions of the cervix (SIL) and cervical cancer (CC), and evaluated the level of mRNA expression of *IL10* (mRNA-IL10) systemically and in the cervix and the IL10 protein level in serum.

## Materials and methods

Using a cross-sectional design, samples of peripheral blood of patients with SIL (*n* = 204), patients with CC (*n* = 80) and patients without SIL (*n* = 166) were used to evaluate SNPs at loci -592A/C (rs1800872), -819C/T (rs1800871), -1082A/G (rs1800896) and -1352A/G (rs1800893) by allelic discrimination with Taqman probes and evaluating the mRNA-IL10. Cervical swabs in women without SIL and cervical biopsies in women with SIL and CC were used for HPV typing and evaluation of mRNA-IL10. Gene expression levels were evaluated by real-time PCR. The genotype and allele frequencies of polymorphisms were analyzed using logistic regression, adjusting for age and genotype of HPV, to determine the association with SIL and CC.

## Results

No significant differences were found between the frequencies of genotypes at loci -819, -1082 and -1352. Individuals who carried at least one copy of the risk allele A of SNP -592, showed an odds ratio (OR) of 2.02 (95% CI 1.26 to 3.25, *P*<0.003) for SIL, an OR of 1.70 (95% CI 1.06 to 2.71, *P*<0.02) for CC, and higher levels of IL10 systemically and in the cervix. Thus, a copy of the risk allele A is sufficient to increase the risk of SIL and CC. We found a significant difference in mRNA-IL10 in both systemic and the cervical levels in women with SIL and CC compared with women without SIL, and in the serum IL10 protein (*P* < 0.0001), being higher in patients carrying the risk allele A of SNP -592. The levels of both mRNA-IL10 and IL10 protein were progressively higher with increasing degree of malignancy of the lesion, so that the presence of IL10 can be regarded as a relevant factor for viral persistence and progression of disease.

## Conclusions

The SNP -592 C/A in the *IL10* promoter is associated with increased risk of SIL and CC and can serve as a biomarker predictive of risk for the development of SIL in the cervix and CC in Mexican women, and is associated with the regulation of this cytokine expression in both cervical and systemic level. HPV 16 E6/E7 proteins bind to Sp1 transcription factor and upregulate *IL*10 gene expression though a GGGGCGG consensus sequence located at -596 to -603 of the human *IL10* gene, and the C/A exchange in the SNP -592 results in increased *IL10* gene promoter activity in HPV-transformed cells.
